# A scoping review of empirical research on executive functions and game intelligence in soccer

**DOI:** 10.3389/fpsyg.2025.1536174

**Published:** 2025-03-31

**Authors:** Jan Arvid Haugan, Kathrine Lervold, Hilde Kaalvik, Frode Moen

**Affiliations:** ^1^Department of Teacher Education, Faculty of Social and Educational Sciences, Norwegian University of Science and Technology, Trondheim, Norway; ^2^Statped, Trondheim, Norway; ^3^Centre for Elite Sports Research, Department of Education and Lifelong Learning, Faculty of Social and Educational Sciences, Norwegian University of Science and Technology, Trondheim, Norway

**Keywords:** soccer, executive functions, game intelligence, elite, review

## Abstract

**Introduction:**

Executive functions (EFs)—including working memory, cognitive flexibility, inhibitory control, and planning—are essential for adaptive decision-making in dynamic environments like elite soccer. This scoping review explores the relationship between EFs and game intelligence in adult elite soccer players.

**Methods:**

A systematic search was conducted across six major databases: Scopus, Web of Science, SportDiscus, PubMed, PsycInfo, and ERIC. Fifteen peer-reviewed empirical studies published between 2000 and 2023 were identified and analyzed for inclusion.

**Results:**

The review reveals a strong association between EFs and players’ ability to process complex game situations, anticipate opponents’ actions, and make strategic decisions under pressure. Evidence also points to possible variations in EF demands across playing positions. Additionally, several studies suggest that EFs may be trainable through perceptual-cognitive interventions, although this area remains underexplored.

**Discussion:**

Despite promising findings, the studies exhibit substantial methodological heterogeneity, particularly in the operationalization of both EFs and game intelligence. This variability limits the comparability and generalizability of results. The review underscores the need for more standardized methodologies, longitudinal research designs, and integrative approaches that account for both cognitive and personality factors to better understand elite soccer performance.

## 1 Introduction

Soccer is claimed to be the most widespread sport in the world (Khaitovich, 2023; Kirkendall, 2020), with participants from both sexes and all skill levels participate in soccer, from children at grassroots levels, to adolescent players, to the highest levels among professional players, and to middle-aged and older adults (Temple and Crane, 2018; Zhang and Pitts, 2018). Thus, soccer has, arguably, the largest number of worldwide players out of all sports and soccer is therefore claimed to be deeply embedded in social contexts (Hyndman et al., 2024; Tovar, 2021). Not surprisingly, soccer is also the most studied research topic among all the sports in the world (Kirkendall, 2020; Sarmento et al., 2018).

The far most reported research topics in soccer research that historically have garnered most interest are topics related to injuries and illnesses (Krustrup and Parnell, 2019; Owoeye et al., 2020). Any injuries, knee injuries, ankle injuries, strain injuries, head injuries, hamstring injuries, and hip & groin injuries are topics that have garnered the most interest within injuries and illnesses (Kirkendall, 2020; Sanchez-Sanchez et al., 2024). Training and performance have also garnered interest among soccer researchers (Clemente et al., 2021; Nicholson et al., 2022). The work to investigate match performance has been of special interest, and how match performance is associated to the physical demands in soccer (Armendáriz et al., 2023; Julian et al., 2021). Physical demands such as strength, endurance, agility and anaerobic capacity have been of special interest (Thapa et al., 2022; van de Hoef et al., 2020). Thus, the most popular topics in soccer research are injury or injuries, training and performance, and sport physiology in general.

However, the key idea of soccer is the soccer game and the play-counter play between the two teams and their players, where both teams are trying to outplay the other team. Thus, besides the physical demands associated with soccer, the players are also continually challenged to make decisions in relation to own team players, the team players from the opponent team, and spaces that occurs and closes on the soccer field. The overall aim is to outplay the opponent team by scoring more goals than the opponent. Soccer games and soccer specific training sessions are therefore cognitively highly demanding because of the consistent shifting environment on the soccer field and decisions that need to be made by the players to outplay the opponent team. Thus, soccer games are mentally demanding, and the functions in the brain that regulate the dynamics of the players’ cognitions and actions are of decisive importance. These executive functions are a set of general-purpose control mechanisms that are linked to the pre-frontal cortex of the brain (Miyake and Friedman, 2012; Strauss et al., 2006). These executive functions in the brain can be closely connected to the concept of game intelligence in soccer.

### 1.1 Executive functions and game intelligence

Executive functions (EFs), comprising working memory: the capacity to hold and manipulate information in real time, inhibitory control: the ability to suppress impulsive responses and focus on relevant stimuli, cognitive flexibility: the ability to switch between tasks and adapt to new rules or situations, and planning: the ability to anticipate future scenarios and organize goal-directed behavior, are key cognitive processes that underpin complex decision-making and adaptive behaviors in sport contexts (Diamond, 2013; Heilmann et al., 2022). In elite soccer, planning supports players in devising strategic actions, predicting opponents’ behaviors, and coordinating movements with teammates, which are critical for optimal tactical executions during matches.

Game intelligence in soccer can be defined as the ability to perceive, interpret, and predict relevant patterns of play in highly dynamic environments, and to make rapid, adaptive decisions that optimize team performance (Roca et al., 2013; Teoldo et al., 2021). This concept encompasses not only executive functions (EFs), such as working memory, cognitive flexibility, and inhibitory control, but also perceptual-cognitive skills, including pattern recognition, visual search behaviors, and anticipatory decision-making (Mann et al., 2007; Vestberg et al., 2012). According to Habekost et al. (2024), cognition in elite soccer relies on the dynamic interplay between attentional control, perceptual processing, and action planning, which together support players’ abilities to identify situational affordances and execute contextually appropriate actions. Thus, *game intelligence* can be understood as an integrated cognitive capacity where EFs regulate higher-order decision processes and enable players to “read the game” and anticipate opponent behavior.

This dual-process perspective highlights the importance of studying both domain-general executive functions and domain-specific perceptual-cognitive abilities when examining elite soccer performance. Recent research has further expanded this understanding by highlighting how executive functions interact with personality traits to shape the cognitive profiles of elite players. Bonetti et al. (2025) demonstrated that elite soccer players exhibit distinctive combinations of cognitive and personality characteristics, suggesting that psychological factors such as resilience, openness, and emotional stability may complement executive functions to support high-level performance. These findings indicate that game intelligence is likely not solely dependent on cognitive mechanisms, but also on stable personality dimensions that influence players’ decision-making and adaptability under pressure.

First, the game of soccer put high demands on the players attentional resources because of the constant shifting contextual environment on the soccer field (Vestberg et al., 2012). Attentional resources can be divided into updating, shifting, inhibition and perceptual anticipation (Miyake and Friedman, 2012). Updating is the ability to constantly monitor the soccer field and add and delete the content of working memory. Shifting is the ability soccer players need to be flexible and switch their attention between different tasks and mental sets. As an example, soccer players must use their attentional resources to handle the ball when they receive it and monitor different spaces in the field for teammates to pass the ball to in the next moment. Inhibition is the ability to override dominant or prepotent responses in the situation, as when a player is tempted to pass the ball to the first and most obvious movement of a teammate, and the best action is to pass the ball to a teammate who is not so visible for the opponent team. The player must be able to quickly adapt, change strategy and inhibit the dominant response. Perceptual anticipation is the ability to understand what will happen next, based on the information the player has in the present moment. Therefore, it is claimed that good soccer players have excellent spatial attention, can use their attention flexible to detect important information in the soccer field and read the game so they can predict what will happen in the play-counter play (Vestberg et al., 2012).

Thus, excellent soccer players are able to make optimal decisions based on the movements of teammates and opponents that are favorable in the play-counter play interaction against the opponent team. The ability to anticipate the play-counter play can be specified by the attentional-, perceptual- and cognitive processing that occurs when players make decisions and execute actions based on how they perceive the current situation on the soccer field (Jordet et al., 2020). The most important environmental information from the soccer field is perceived by the player and placed in the player’s working memory, and that information is processed and interpreted by a comparison with learned actions from similar situations stored in long time memory (Moen et al., 2018). An effective perceptual-cognitive process requires that the player has developed actions that are suitable in the situation that occurs on the soccer field (Appelbaum and Erickson, 2018; Mangine et al., 2014). Thus, attentional resources in combination with perceptual-cognitive skills are crucial for performances in dynamic sports such as soccer (Mann et al., 2007). The soccer player’s ability to execute effective actions in the play-counter play interaction is defined as game intelligence (Furley and Memmert, 2013).

In sum, game intelligence is closely associated with executive functions and refers to the ability to execute appropriate decisions and effective actions in the soccer game (Mann et al., 2019; Williams and Jackson, 2019). Interestingly, tactical skills such as perceptual-cognitive skills and game intelligence are in recent years claimed to be valid predictors of future success in soccer (Huijgen et al., 2014; Murr et al., 2018; Roca et al., 2018) and are therefore highlighted as especially important among coaches in talent identification (Larkin and O’Connor, 2017; Bergkamp et al., 2021; Roberts et al., 2019).

### 1.2 The present study

Studies have utilized various methods to measure game intelligence and executive functioning, including video-based decision-making tasks: These assessments present players with real or simulated game scenarios to evaluate their decision-making (Ward and Williams, 2003). In-game performance analysis: Observational studies assess players’ tactical awareness and decisions during actual games (Williams and Hodges, 2005). Simulation-based assessments: Recent advances in virtual reality (VR) have enabled more immersive tests of game intelligence, such as the use of 3D simulations to replicate complex in-game scenarios (Romeas et al., 2016). Systematic reviews have offered valuable insights into this intersection, synthesizing findings from empirical studies to provide a comprehensive understanding of the relationship between game intelligence and executive functions (e.g., Brimmell et al., 2022; Furley et al., 2023; Kalén et al., 2021; Scharfen and Memmert, 2019; Zhao et al., 2022). However, research on game intelligence in soccer seems scarce, and the mental capacities associated to the play-interplay process in soccer need further exploration among researchers. Most studies have explored youth level players, and elite level players are understudied. The aim of the current study is to map and explore the extent, range and nature of the existing literature on game intelligence in soccer to identify research gaps and suggest a research agenda, based on the following research question: “What does research between 2000 and 2023 on adult elite level players reveals about the interrelationship between executive functions and game intelligence in soccer?”

## 2 Methods

This scoping review was conducted following Arksey and O'Malley’s (2005) six-step framework, aiming to map the existing empirical research on the relationship between executive functions (EFs) and game intelligence in adult elite soccer players. Given the exploratory stage of this research field and the diversity of study designs and conceptualizations involved, a scoping review methodology was deemed appropriate.

Studies were included if they investigated adult elite soccer players aged 18 years or older, competing at national or international top levels. To be eligible, studies needed to examine either executive functions—such as working memory, cognitive flexibility, inhibitory control and perceptual anticipation—or cognitive processes closely linked to executive functioning within the context of game intelligence. This included research on perceptual-cognitive skills like visual search behavior, anticipation, and tactical decision-making, which are understood to contribute to players’ situational awareness and adaptive performance in complex, dynamic environments. Furthermore, studies had to address aspects of game intelligence, which in this review is defined as the integrated use of executive functions, perceptual skills, and decision-making abilities that enable players to interpret, anticipate, and respond effectively to evolving patterns of play. Eligible studies were required to be empirical, peer-reviewed, and published in English between January 1st, 2000, and December 31st, 2023.

The inclusion of studies focusing on visual search behavior is justified by the understanding that attentional control and perceptual processing are fundamental components of executive functioning during soccer performance. These processes support core executive functions such as working memory updating, cognitive flexibility, inhibition and perceptual anticipation, which together enable players to manage complex tactical demands and effectively “read the game” (Mann et al., 2007). Similarly, studies focusing on injury prevention were included if they explored the role of executive functions in mitigating cognitive errors and attentional lapses that may influence both injury risk and game-related decision-making, as demonstrated by Scharfen and Memmert (2021a,b).

A comprehensive search was conducted in March 2022 across the databases Scopus, Web of Science, SportDiscus, PubMed, PsycInfo, and ERIC. The search strategy combined terms related to soccer, executive functions, and game intelligence, and the complete search string is presented in Table 1. Following the removal of duplicates, two authors independently screened titles and abstracts, after which full-text screening was conducted based on the defined inclusion criteria. The initial search yielded 958 records, of which 12 studies met the inclusion criteria after full-text review. Through backward citation tracking and snowballing, an additional three studies were identified, resulting in a total of 15 studies included in the review.

**Table 1:** Keywords facilitating searches.

	Concept A: Soccer (and associated terms)	Concept B: Game intelligence (and associated terms)	Concept C: Elite level (and associated terms)
↑OR↓	Search terms used: “Soccer” “Football”	Search terms used: “Intelligen*” “Game intelligen*” “Sport intelligen*” “Soccer intelligen*” “Football intelligen*” “Tactic*” “Tactical knowledge*” “Tactical performance*”	Search terms used: “Elite*” “Elite football*” “Elite soccer*” “Professional*” “Professional football*” “Professional soccer*”
	←AND→		

**Figure 1:**
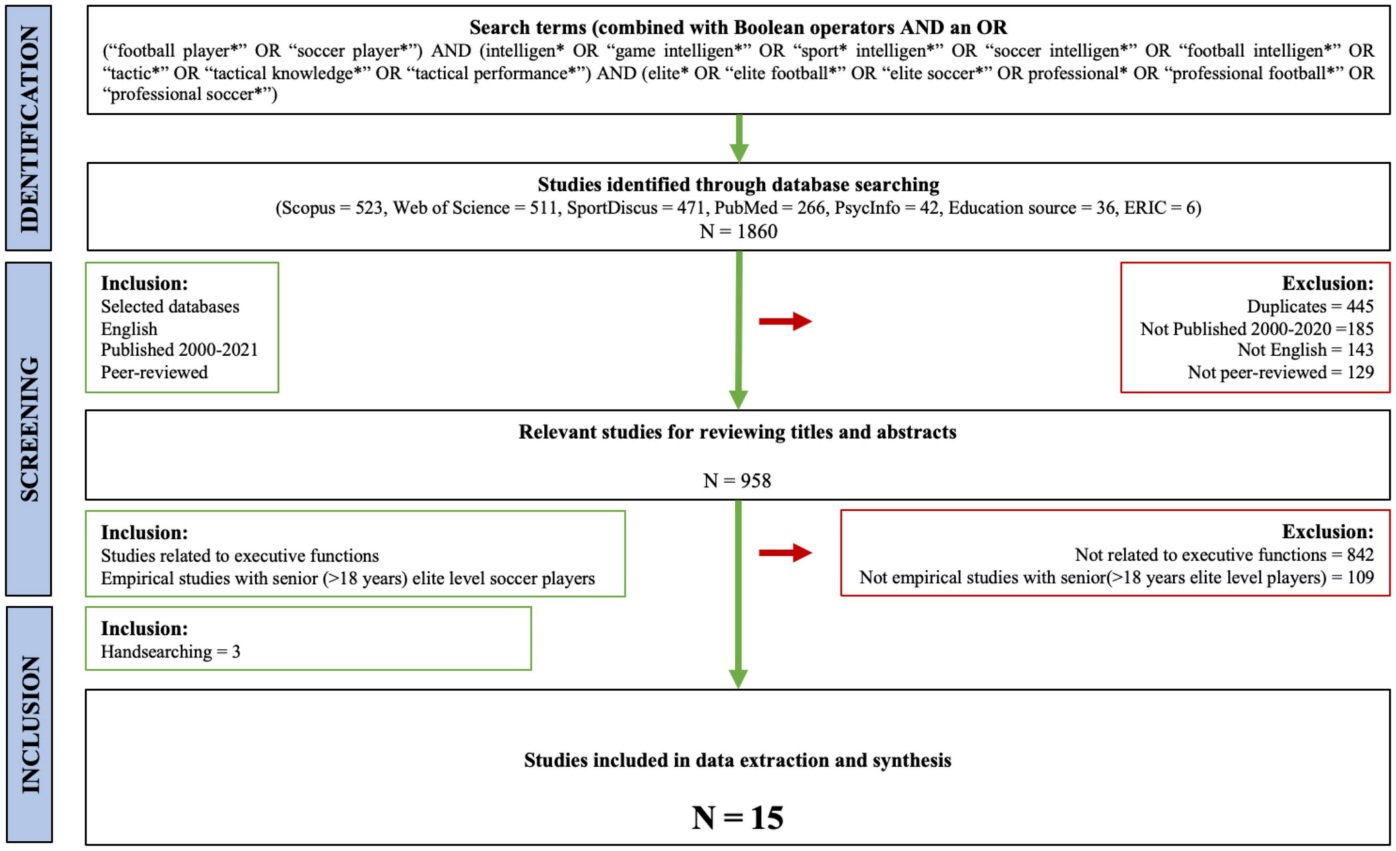
The scoping process (inspired by Moher et al., 2010).

First, the research question was defined. The search strategy was designed to find studies primarily discussing football players’ game intelligence in elite football. We used a modified versin of a PICO framework as aid in designing a suitable research question which formed the basis for the review protocol, the search strategy, inclusion and exclusion criteria and data extraction. In the next steps, relevant studies were identified, selected and charted. The scoping process is illustrated in Figure 1.

### 2.1 Identifying relevant studies

Figure 1 illustrates the methodological identification and screening process of selecting relevant articles for the scoping review. The keywords for our search in databases were based on previous studies on the phenomena and consultations with experts in the field. Based on the research question, different synonyms for soccer and game intelligence emerged. These keywords constituted the basis for scoping the field of relevant studies. Table 1 presents how the search terms were combined into a search string using the Boolean operators AND and OR. The database search conducted March 2022 in Scopus, Web of Science, SportDiscus, PubMed, PsycInfo, Eric, Education source resulted in 958 articles that were transferred to the bibliographic software EndNote.

The process started with delineating the search to studies written in English that were published between January 1st, 2000 and December 31st, 2023. To ensure methodological quality, only peer-reviewed studies were included.

### 2.2 Screening

After importing the references in EndNote and removal of duplicates, the search identified 958 references, which could be potentially relevant. The first and second author independently screened titles and abstracts, followed by a full text screening to determine eligibility. First, results that were not published between 2000 and 2023, not in English, or not peer-reviewed, were excluded. Second, studies not related to executive functions or adult elite players were removed.

In total, 12 studies focused on game intelligence among adult elite level soccer players. To identify potential studies not included or potentially missed in the database search we snowballed and hand searched for relevant studies in previous reviews and in the articles selected for this scoping review, this resulted in three additional studies, and a total of 15 studies were included in the review.

### 2.3 Studies included in data extraction and synthesis

A total of 15 studies met the inclusion criteria for further analysis (see Table 2).

**Table 2:** Description of the content in the included studies.

Author(s)	Title	Objective(s)	Participants	Methods	Results	Conclusions
Beavan et al. (2020)	The rise and fall of executive functions in high-level football players	This study investigated how executive functions (EFs) develop across the lifespan of high-level football players, focusing on the influence of age and playing experience. The research aimed to identify developmental patterns in EFs, determine critical periods for cognitive growth, and evaluate whether these abilities can serve as reliable indicators for football performance.	The study involved 343 male football players aged 10.34 to 34.72 years, representing U12 to senior levels of a professional German football club. Data was collected from 1,018 observations over three seasons, with players providing 1–5 data points each.	Four cognitive tasks were administered to assess key EF components: 1. Precued choice reaction-time task for processing speed and attention. 2 Stop-signal reaction-time task for inhibitory control. 3. Sustained attention task for vigilance and consistency. 4. Multiple-object tracking task for tracking dynamic stimuli and spatial awareness. The dependent variables derived included response times and accuracy measures. Linear and non-linear mixed-effects regression models analyzed the relationships between EF, age, experience, and positional differences.	Developmental Trajectory: The results indicated a negatively accelerated developmental curve for EFs, with rapid improvements during childhood and adolescence, followed by a plateau around the age of 21. This pattern aligns with the general population’s cognitive maturation trends, suggesting that EFs in football players are primarily influenced by age-related neurological development. Experience: Playing experience exhibited a limited impact on EF performance. While some improvements were linked to increased exposure to the game, these accounted for only a small portion of variance in EF scores. Positional Differences: Positional effects were minimal, except for sustained attention, where an interaction between age and position was observed. Defensive players showed slightly greater improvements in sustained attention accuracy and response times over time.	The findings reveal that EF development in football players mirrors natural cognitive growth in the general population, rather than being uniquely shaped by sport-specific training or experience. These results cast doubt on the validity of using EFs as standalone predictors of football performance potential, particularly in contexts where player development and training focus heavily on physical and technical skills. The study suggests that while EFs are important for in-game decision-making and tactical adaptability, they may not be as critical for distinguishing between talent levels as previously thought
Ilić (2016)	Structures and differences in cognitive abilities of top handball, volleyball, basketball, and soccer players	This study aimed to compare cognitive abilities across athletes in four sports: handball, volleyball, basketball, and soccer. The primary focus was to identify specific cognitive structures and highlight differences that arise due to the distinct tactical and physical demands of each sport.	The sample consisted of 200 male athletes, with 50 participants from each sport. All participants were elite-level players, competing at the highest levels of their respective sports.	The KOG 3 test battery was employed to measure cognitive abilities across three domains: 1. Input processing: Quick observation and environmental awareness. 2. Serial processing: Logical sequencing and decision-making. 3. Parallel processing: Simultaneous information integration and problem-solving. Principal component analysis identified cognitive structures, while canonical discriminant analysis evaluated inter-group differences.	General Findings: A single general cognitive factor was identified across all athletes. Volleyball players achieved the highest average scores in all cognitive tests, followed by basketball, handball, and soccer players. Processor Efficiency: Input processor (IT-1) showed the highest significance for all groups, with volleyball and basketball players excelling. Parallel processor (S-1) scores indicated that volleyball and basketball players had stronger visual–spatial reasoning, attributed to the tactical and dynamic demands of these sports. Discriminant Analysis: One discriminant function distinguished basketball and volleyball players from others, emphasizing the importance of perception and spatial processing in smaller court-based sports.	Cognitive abilities, particularly perceptual reasoning and visual–spatial skills, are crucial for success in dynamic team sports. Volleyball and basketball impose greater cognitive demands, reflecting their need for rapid perception and tactical decision-making in confined spaces. These findings underline the importance of cognitive testing in athlete selection and suggest tailoring training to enhance specific cognitive abilities relevant to each sport.
Kermarrec and Bossard (2020)	Defensive Soccer Players` Decision Making: A Naturalistic Study	To describe the decision making of professional defensive football players according to the recognition-primed decision (RPD) model and to investigate relationships between their recognition processes and use of salient situational features in a naturalistic setting.	Four international level male football players (mean age: 25.5 years, SD = 3.8 years)	Behavioral data were obtained from video recordings of four high-level football players in a top-level game, supplemented by verbal data collected during video-cued recall interviews, with the players watching the videotapes. Seven critical defensive stages in one game were studied. The data were analyzed using content analysis in relation to the RPD model in five steps.	Considering the salient features the athletes used, 112 decisions were classified into three types of processes and eight typical decisions for defensive stages. These findings are discussed from three perspectives: the salient features the athletes used to assess the situation, the recognition processes of expert sport players’ decision making, and future directions for research and decision-making training.	An original contribution from NDM for sport psychology should consist of studying decision making, by combining a description of refined and significant elements of the course of action, while identifying under- lying mechanisms of situation recognition.
Klatt and Nerb (2021)	Position-Specific Attentional Skills in Team Sports: A Comparison between Defensive and Offensive Football Players	This study explored whether players in different football positions—offensive versus defensive—demonstrate distinct attentional skills, particularly regarding in attentional blindness. The research focused on identifying position-specific cognitive advantages and their implications for game performance and tactical adaptability.	61 participants (35 experts, Mean age = 24.6 years, SD = 4.0 years, 26 amateurs, Mean age = 24.9 years, SD = 4.5 years) was included in the analysis	Three experiments were conducted using in attentional blindness paradigms: Experiment 1: Participants counted basketball passes while an unexpected, non-sport-specific object appeared. This assessed general in attentional blindness. Experiment 2: The same task was repeated with a focus on positional differences, comparing noticing rates among various football roles. Experiment 3: A football-specific task required participants to identify tactical solutions in game scenarios, introducing unexpected free-standing players to measure in attentional blindness in a sport-relevant context. Noticing rates for unexpected objects and their relevance to tactical decision-making were analyzed.	Experiment 1: Offensive players, particularly strikers, exhibited higher noticing rates for unexpected objects compared to defensive players. This indicated a general attentional advantage for offensive roles. Experiment 2: A more detailed analysis confirmed that offensive players consistently outperformed defensive players in noticing unexpected objects. Strikers demonstrated the greatest awareness, likely due to their role’s reliance on rapid situational assessment and spatial awareness. Experiment 3: Offensive midfielders excelled in detecting unmarked players in tactical scenarios, highlighting their ability to perceive game-relevant stimuli. Defensive players exhibited higher rates of in attentional blindness, potentially reflecting their focus on immediate threats rather than peripheral awareness.	Attentional skills are highly position-dependent in football. Offensive players, particularly strikers and offensive midfielders, possess superior awareness of unexpected stimuli, aiding their adaptability and decision-making in dynamic game situations. Defensive players’ higher in attentional blindness suggests a more narrowed focus on specific tasks, such as marking opponents. These findings have significant implications for training, as coaches can tailor cognitive drills to enhance position-specific skills, fostering both individual and team performance.
Krupitzer et al. (2022)	CortexVR: Immersive analysis and training for cognitive executive functions of soccer players using virtual reality and machine learning	The study aimed to develop and evaluate CortexVR, an immersive virtual reality (VR) system for analyzing and training executive functions (EFs) in soccer players. The system leverages VR and machine learning to provide a scalable, immersive environment to enhance key cognitive skills, such as working memory, cognitive flexibility, and inhibition	Sample Size: 37 participants, aged 21–35 years, including 25 males and 12 females. Experience: Some participants had prior experience with VR or gaming, while others were first-time users. Context: The study was conducted in collaboration with TSG Hoffenheim, a professional soccer club in Germany.	System Description: CortexVR incorporates realistic game situations and movements based on Bundesliga tracking data. The system includes three game modes: Tracking Players, Count Players, and Find Ball, each targeting specific EFs. A Coach App was developed to configure training sessions and analyze user performance (e.g., reaction times and accuracy). Experimental Setup: Participants used both a VR-based system (Oculus Rift) and a non-VR desktop version. Each participant played three game modes with both systems to assess usability and user experience. Data was collected on Effort Expectancy (ease of use) and Hedonic Motivation (enjoyment). Evaluation: A reinforcement learning algorithm adapted game difficulty in real-time based on user performance. Both technical performance (e.g., latency) and user feedback were analyzed.	Technical Performance: The VR system demonstrated acceptable motion-to-photon latency (~34.4 ms) and high frame rates (~78 FPS), ensuring smooth performance without negatively impacting the user experience. User Feedback: VR significantly improved Hedonic Motivation, with participants reporting greater enjoyment and a better sense of immersion compared to the desktop version. Effort Expectancy scores for VR and non-VR setups were comparable, indicating that VR did not impose a higher cognitive load despite being a novel interaction paradigm for some participants. Game Modes: The Tracking Players mode provided the most effective training for working memory. The Find Ball mode emphasized cognitive flexibility by requiring participants to infer ball positions based on player formations. Adaptive Learning: The reinforcement learning system effectively adjusted game difficulty based on user performance, with a cumulative increase in rewards over time.	Enhanced Training Efficiency: CortexVR provided an immersive and distraction-free environment for EF training, demonstrating its potential to replace or supplement traditional EF training systems. Scalability and Accessibility: The system’s modular design and cost-effectiveness make it viable for widespread adoption in professional soccer clubs and academies. Future Directions: Longitudinal studies are needed to evaluate the sustained impact of CortexVR on player performance. Refinement of game modes to incorporate specific soccer scenarios (e.g., corner kicks, set plays) would further enhance relevance and applicability.
Nunez et al. (2009)	Differences between expert and novice soccer players when using movement precues to shoot a penalty kick.	This study explored the influence of visual cues and anticipation mechanisms in soccer penalty scenarios. It examined the decision-making processes of goalkeepers and kickers, focusing on response timing and pre-cue utilization to improve performance efficiency.	Twenty male participants were categorized into two groups: expert players (10) with at least 10 years of semi-professional experience and novices (10) with limited soccer exposure. The average ages were 25.7 years for experts and 22.1 years for novices.	Experimental Setup: Participants interacted with a simulation system displaying penalty scenarios on a large screen. An eye-tracking device recorded visual focus and response times. Tasks: Players were asked to predict and respond to penalty directions using pre-cue movements, such as the kicker’s non-kicking leg. Each participant completed 30 penalty kicks per session.	Experts exhibited higher accuracy in predicting the direction and height of penalty shots compared to novices. Response times were faster in experts, particularly in initiating movements based on pre-cues. Experts focused predominantly on the non-kicking leg and ball trajectory, while novices distributed their attention broadly across irrelevant cues.	The study highlighted the importance of anticipatory visual search strategies and pre-cue utilization in expert goalkeepers, suggesting that targeted training on these aspects can significantly enhance performance.
Nunez et al. (2010)	Effects of providing advance cues during a soccer penalty kick on the kicker’s rate of success.	This research analyzed the impact of providing explicit pre-cue information on the success rates of penalty kicks. The study investigated how such cues improve decision-making and ball placement accuracy.	Thirty-two male professional soccer players, divided into four groups: experimental, discovery, placebo, and control, with a mean age of 23.2 years and over 10,000 h of practice experience.	The Preindex Trainer system was employed to present pre-cue information about goalkeeper movements during penalties. Participants underwent pretests, training sessions, and post-tests over a seven-day period, completing multiple penalty kicks. Data were analyzed using a mixed factorial design to assess success rates, decision times, and ball trajectory.	Players in the experimental group, who received explicit pre-cues, showed significantly higher success rates (83.1%). The benefit of pre-cues persisted across retests after 24 h and 7 days, indicating retention of anticipatory skills. Discovery group participants improved their implicit understanding of pre-cues, while placebo and control groups showed no significant changes.	Explicit pre-cues substantially enhance penalty performance by improving players’ ability to anticipate goalkeeper movements. Integrating such cues into training can refine perceptual decision-making.
Savelsbergh et al. (2002)	Visual search, anticipation and expertise in soccer goalkeepers	The study investigated the anticipatory skills of expert versus novice goalkeepers during penalty kicks, focusing on visual search efficiency and decision-making.	Fourteen goalkeepers, seven experts and seven novices, were recruited. Experts had a minimum of 10 years of semi-professional experience.	Penalty scenarios were presented via video clips, and participants predicted ball trajectory using a joystick. Eye-tracking technology recorded fixations, while response accuracy and timing were analyzed.	Experts saved more penalties and displayed longer fixations on key areas like the kicking leg and ball, while novices fixated on irrelevant cues. Experts initiated responses later, reflecting superior anticipatory processing.	The study underscores the importance of refined visual search behavior in expert goalkeepers. Anticipatory skills can be improved through training focused on critical visual cues.
Savelsbergh et al. (2005)	Anticipation and visual search behavior in expert soccer goalkeepers.	This study explored visual search behavior and anticipation in expert soccer goalkeepers during penalty kicks. It aimed to identify intra-group differences among successful and unsuccessful goalkeepers.	Sixteen professional goalkeepers from top Dutch leagues were divided into successful and unsuccessful groups based on penalty save rates.	Participants viewed penalty scenarios on a large screen and responded using a joystick to predict shot direction. Eye-tracking technology recorded visual fixations, durations, and search strategies.	Successful goalkeepers were more accurate in predicting shot height and direction, displaying longer fixations on critical cues like the non-kicking leg. Unsuccessful goalkeepers tended to fixate on less predictive areas, such as the trunk and arms. Anticipatory response times were delayed in successful goalkeepers, allowing more comprehensive information processing.	Efficient visual search strategies, characterized by fewer but longer fixations on key cues, distinguish successful goalkeepers. Training programs should emphasize these strategies to improve anticipatory performance.
Radke et al. (2023)	Being ahead of the game - the association between executive functions and football performance in high-level football players	The study investigated the relationship between executive functions (EFs) and football performance across different age groups and playing positions. By using both subjective (coach ratings) and objective (Footbonaut score) performance measures, the research aimed to explore how core EF components influence tactical and technical abilities.	Sample size: 176 male football players aged 10.8–37.1 years. Context: All participants were members of a professional German fist-division club, ranging from U12 to U23 teams. Inclusion criteria: Players participated in regular diagnostics, which included EF and football perfomance assessments	Three EF subcomponents were assessed: Cognitive flexibility: Ability to adapt to changing game demands. Inhibition: Suppression of impulsive actions. Working memory: Retaining and processing game-relevant information. Performance measures included: Subjective: Coach ratings of game intelligence and adaptability. Objective: Footbonaut scores, a task assessing accuracy and speed in passing, particularly relevant for defenders and goalkeepers. Statistical models analyzed the associations between EFs and performance metrics.	Cognitive Flexibility: Showed the strongest correlation with game intelligence and Footbonaut scores, highlighting its critical role in adapting to dynamic match conditions. Inhibition and Working Memory: While significant, these components were less strongly linked to performance, suggesting they are secondary to cognitive flexibility in high-pressure scenarios. Positional Differences: Footbonaut scores revealed nuanced differences, with defenders and goalkeepers benefitting from tailored EF assessments due to their unique tactical roles.	EFs, particularly cognitive flexibility, are vital for football success, influencing both subjective coach ratings and objective performance metrics. The study emphasizes the value of integrating EF training into player development programs, with a focus on enhancing adaptability and tactical awareness. By tailoring assessments to positional demands, clubs can optimize talent identification and training strategies, ensuring players are cognitively equipped for high-level competition.
Scharfen and Memmert (2021a,b)	Fundamental relationships of executive functions and physiological abilities with game intelligence, game time and injuries in elite soccer players	This study aimed to examine the relationships between executive functions (EFs), physiological abilities, and key soccer performance outcomes, including game intelligence, game time, and injury rates. The research sought to determine how cognitive and physical skills collectively contribute to success across age groups in elite soccer.	A total of 172 elite soccer players, aged 12–34 years, participated. These included youth and senior players competing at professional and academy levels	Participants completed assessments of: Executive Functions: Tests included multiple-object tracking (spatial awareness), working memory capacity (WMC), cognitive flexibility (CF), and inhibition (impulse control). Physiological Abilities: Physical performance was measured through sprinting, jumping, and endurance tests. Performance Metrics: Game intelligence was evaluated through coach ratings, while game time and injury data were collected longitudinally. Correlation and regression analyses explored the interplay between cognitive and physical abilities and their effects on performance outcomes.	Executive Functions and Game Intelligence: WMC, CF, and overall EF scores were strongly correlated with game intelligence, underscoring their role in tactical decision-making and situational awareness. Game Time: Selective attention, sprinting ability, and endurance were key predictors of playing time across all age groups. Players with higher EF scores also logged more game time, likely due to superior adaptability and anticipation skills. Injuries: Sprinting and endurance metrics were linked to non-contact injuries, while contact injuries showed weaker associations with physical or cognitive measures. Age and Development: The importance of EFs and physical abilities varied with age, with younger players relying more on physical attributes, while cognitive skills became increasingly critical in older age groups.	The study highlights the multifaceted nature of soccer performance, emphasizing the synergy between EFs and physiological abilities. EFs, particularly WMC and CF, are foundational for game intelligence and playing time, making them essential components of talent prediction models. However, physical abilities remain crucial for mitigating injury risks and maintaining on-field effectiveness. Training programs should integrate cognitive and physical development, tailored to the age and positional demands of players, to maximize their potential and reduce injury risks.
Šetić et al. (2017)	Validation study of the tactical, technical and social competencies of football players scale	The study aimed to validate a new scale designed to assess tactical, technical, and social competencies in football players. The goal was to create a reliable tool for evaluating players’ multidimensional skills and aiding coaches in talent identification and development.	Overall 166 football players (*N* = 81 seniors and 85 juniors) from several BiH Premier League clubs took part in the study. The average age of participants was M = 21.14 (SD = 4.91); the average age for junior players was M = 17.23 (SD = 0.49), while for seniors it was M = 25.15 (SD = 4.21).	The study employed a mixed-methods approach: Scale Development: The scale included items assessing tactical (e.g., decision-making), technical (e.g., ball control), and social (e.g., teamwork) competencies. Validation: Statistical analyses evaluated the scale’s reliability and validity, including factor analysis and inter-rater reliability measures. Coach Feedback: Coaches provided qualitative and quantitative feedback on the scale’s relevance and usability.	Factor Analysis: The scale demonstrated a clear three-factor structure corresponding to tactical, technical, and social competencies. Each factor showed high internal consistency. Reliability: Inter-rater reliability scores were strong, indicating that coaches consistently rated players’ competencies. Practical Application: Coaches reported that the scale provided actionable insights for identifying strengths and areas for improvement in players, particularly in tactical awareness and teamwork.	The validated scale offers a robust tool for assessing football players’ tactical, technical, and social competencies. Its applicability across competitive levels makes it valuable for coaches seeking to develop well-rounded players. Future research should explore the scale’s predictive validity in relation to long-term player performance and career progression.
Šetić et al. (2019)	Validation scale study for assessment of tactical and technical competencies among footballers - assessment by trainers	The study sought to validate a scale for assessing tactical and technical competencies among football players. The scale aimed to help coaches evaluate and compare players’ skills comprehensively, enabling effective talent development and strategic training.	165 players from Premier League Bosnia and Herzegovina. The average age of the participants is 21.14 ± 4.91; The average age of the junior is 17.23 ± 0.49, while the average age of the senior is 25.15 ± 4.21.	Scale Design: The scale was constructed to measure: Tactical Competencies: Decision-making, positioning, and awareness. Technical Competencies: Ball control, passing, shooting, and dribbling. Validation Process: The study utilized statistical methods, including factor analysis, to determine the scale’s structure and reliability. Inter-rater consistency was assessed to ensure that coaches could use the scale consistently. Implementation: Coaches applied the scale during matches and training sessions, providing data for analysis.	Factor Analysis: Results confirmed a two-factor structure (tactical and technical competencies), demonstrating high internal consistency for both components. Reliability: Inter-rater reliability was strong, indicating that different coaches rated players similarly when using the scale. Practical Insights: The scale allowed coaches to identify specific strengths and weaknesses in players, particularly in tactical decision-making and technical execution.	The validated scale provides a reliable and user-friendly tool for assessing tactical and technical competencies in football players. By enabling coaches to evaluate players holistically, the scale supports targeted training interventions and effective talent identification. Future research could investigate its predictive validity for long-term player performance and professional success.
Vestberg et al. (2012)	Executive Functions Predict the Success of Top-Soccer Players	The study aimed to determine whether general executive functions (EFs) could predict soccer success. By examining EFs such as cognitive flexibility, response inhibition, and creativity, the research sought to establish their importance in player performance and talent identification.	57 male (*n* = 31) and female (*n* = 26) players (Table 1). 14 male and 15 female participants from the Swedish highest national soccer leagues (Allsvenskan) were included in the highest division group, HD (Mage = 25.3; SD: 4.2). 17 male participants playing in the Swedish 3rd national division (called Division 1) and 11 female participants from Swedish 2nd national division were included in the lower division group, LD (Mage = 22.8; SD: 4.1).	EF Assessment: Standardized neuropsychological tools measured core EF components like cognitive flexibility, inhibition, and multitasking ability. Comparative Analysis: EF scores were compared across HD players, LD players, and the norm group. Performance Metrics: Player success was evaluated based on goals, assists, and other game-related statistics over two subsequent seasons.	Group Comparisons: Both HD and LD players scored significantly higher on EF tests compared to the norm group, with HD players outperforming LD players. Correlation with Success: EF scores correlated strongly with subsequent on-field performance, including goals and assists. Predictive Value: EF assessments provided reliable predictions of soccer success, particularly in decision-making and game intelligence.	The study demonstrated that EFs are critical predictors of soccer success. High-performing players exhibited superior cognitive flexibility and multitasking abilities, enabling better in-game decisions and tactical execution. These findings support the inclusion of EF assessments in talent identification and development programs, highlighting their value beyond traditional physical and technical evaluations.
Vestberg et al. (2020)	Level of play and coach-rated game intelligence are related to performance on design fluency in elite soccer players	This study examined the relationship between executive functions (EFs), coach-rated game intelligence, and soccer performance. It aimed to identify how EFs influence key metrics like assists and adaptability, particularly among elite players.	Elite soccer players (*N* = 51). The included subjects belonged to the four Swedish premier league teams and consisted of both male (*n* = 19; Mean age = 24.4, SD = 4.73) and female (*n* = 32; Mean age = 24.5, SD = 4.63) players. Out of the 51 players 23 had previously played for fourteen national teams on senior level.	EF Measurement: The Design Fluency (DF) test assessed cognitive flexibility and creativity, key EF components for dynamic problem-solving. Coach Ratings: Game intelligence was rated by coaches based on tactical understanding and decision-making. Performance Metrics: Assists and goals were tracked over a season to evaluate objective performance.	EFs and Game Intelligence: DF scores correlated moderately with coach-rated game intelligence, emphasizing the role of cognitive flexibility in tactical decision-making. Assists vs. Goals: While DF scores correlated with assists, they did not predict goal-scoring, suggesting that creativity and planning are more relevant for setting up plays than finishing them. Performance Differentiation: National team players outperformed premier league players in DF tests, highlighting EFs as distinguishing factors at the highest levels.	EFs, particularly cognitive flexibility, are critical for game intelligence and playmaking success. The findings underscore the importance of cognitive training in elite soccer, with applications for identifying and developing top-level talent. Future research should explore how specific EFs contribute to different aspects of game performance, refining talent development strategies further.

### 2.4 Data analysis

To synthesize the findings from the included studies, we conducted a reflexive thematic analysis (RTA) as outlined by Braun et al. (2023). This approach was selected due to its flexibility in identifying patterns of meaning across heterogeneous studies and its suitability for scoping reviews. The analysis followed a six-phase iterative process, starting with familiarization with the data through repeated readings of the included articles. Initial codes were generated based on the studies’ aims, methods, and key findings related to executive functions and game intelligence. These codes were then organized into candidate themes through an inductive process, allowing for the identification of recurrent patterns without imposing predefined categories.

Themes were refined through constant comparison, discussion among the authors, and critical reflection on our interpretations, ensuring that the thematic structure accurately represented the diversity of the included studies. The final thematic framework consists of seven overarching themes, which are presented in the results section: developmental trajectories of executive functions, the relationship between EFs and soccer performance, position-specific cognitive skills, perceptual-cognitive interventions, visual search behavior and anticipation, the role of EFs in injury prevention, and neuropsychological assessment of executive functions.

By applying reflexive thematic analysis, we aimed to provide a nuanced synthesis that accommodates the methodological diversity of the literature while highlighting the central cognitive processes that underpin game intelligence in elite soccer.

## 3 Results

This scoping review included 15 peer-reviewed empirical studies published between 2000 and 2023, all exploring the relationship between executive functions (EFs) and game intelligence in adult elite soccer players. The included studies show considerable methodological diversity, both in their theoretical frameworks and their approaches to assessing cognitive processes, making synthesis complex but necessary for understanding common patterns and knowledge gaps. While some studies focus on general cognitive capacities measured through standard neuropsychological tasks, others adopt soccer-specific assessments, such as video-based decision-making or perceptual training interventions. Despite this heterogeneity, seven key thematic areas emerged from the analysis: developmental trajectories of executive functions, the relationship between EFs and soccer performance, position-specific cognitive skills, perceptual-cognitive training interventions, visual search behavior and anticipation, the role of EFs in injury prevention, and neuropsychological assessment of executive functions. Below, findings are presented across these themes, with attention to methodological strengths and weaknesses, recurring tendencies, and areas of divergence.

### 3.1 Developmental trajectories of executive function

A recurrent topic in the literature concerns how executive functions develop in elite soccer players over time. Several studies suggest that EFs follow a curvilinear development, with accelerated growth during adolescence and a tendency toward stabilization in early adulthood (Beavan et al., 2020; Verburgh et al., 2014). Beavan et al. (2020) found that the most significant improvements in cognitive flexibility, working memory, and inhibitory control occurred between ages 12 and 20. Beyond this period, gains appeared to level off, possibly reflecting the attainment of a cognitive ceiling related to natural maturation processes and the limits of neuroplasticity in adulthood.

Importantly, these insights are drawn primarily from cross-sectional studies, which limits the ability to draw causal conclusions or track within-individual changes over time. Furthermore, the age categories used in different studies vary, which makes comparison difficult. While some researchers interpret these findings to mean that soccer may selectively attract individuals with naturally higher EF capacities, others propose that prolonged exposure to the cognitive demands of elite soccer environments may help preserve high EF functioning during adulthood (Verburgh et al., 2014).

The field would benefit greatly from longitudinal studies that follow cohorts of players through key developmental windows, allowing researchers to distinguish between maturation effects, training effects, and selection biases. Such studies could clarify whether elite soccer environments contribute to the maintenance or even enhancement of EFs beyond typical developmental trajectories.

### 3.2 Executive functions and soccer performance

Across the literature, EFs emerge as critical cognitive resources for successful soccer performance. Cognitive flexibility, working memory, inhibitory control and perceptual anticipation are particularly highlighted as core mechanisms underpinning in-game decision-making and adaptive tactical behavior (Vestberg et al., 2012; Sakamoto et al., 2018). Players with higher EF capacities consistently demonstrate superior abilities to adjust strategies during dynamic match situations, manage multiple streams of information simultaneously, and suppress irrelevant stimuli to focus on the most critical aspects of play.

Vestberg et al. (2012), for example, linked higher EFs test scores to superior tactical adaptability and successful match outcomes, providing empirical support for the theoretical claim that EFs are foundational to handling the inherent unpredictability of elite-level soccer. However, other studies point to significant limitations, particularly concerning the ecological validity of EFs assessments. Laboratory-based tasks may not fully capture the contextual complexity of match play, where cognitive demands arise in tandem with physical exertion, emotional pressure, and interpersonal coordination.

Another limitation lies in the frequent disconnection between cognitive assessments and objective performance metrics. Few studies integrate EFs testing with granular performance data from matches, such as pass completion rates, defensive actions, or goal contributions. Without such links, the precise influence of EFs on measurable performance outcomes remains difficult to establish. To strengthen this field, future studies should aim to combine cognitive testing with advanced match analytics to better understand how EFs translate into concrete behaviors on the field.

### 3.3 Position-specific cognitive skills

A smaller subset of studies explores the potential differences in EFs demands across playing positions. Research suggests that offensive players, particularly midfielders and forwards, tend to score higher on measures of cognitive flexibility and attentional control than defensive players (Huijgen et al., 2015). This finding aligns with the higher levels of unpredictability, rapid transition, and creativity required in attacking roles, where players must generate and execute complex strategies under tight time constraints.

Conversely, defensive players appear to rely more heavily on anticipatory skills, pattern recognition, and positional discipline. These skills are crucial for reading the game, predicting opponents’ moves, and making preemptive interventions. However, evidence supporting these position-based cognitive profiles is currently limited by small sample sizes and inconsistent classification of playing positions across studies. Furthermore, it remains unclear whether cognitive differences drive players into specific positions or whether the cognitive demands of those positions shape players’ abilities over time.

Clarifying these relationships will require more rigorous and standardized research designs, possibly including longitudinal studies that follow players as they transition across positions or levels of competition. Such work could offer valuable insights into whether cognitive specialization is a product of role adaptation or selection based on inherent cognitive strengths.

### 3.4 Perceptual-cognitive interventions

Research on perceptual-cognitive interventions provides promising, albeit preliminary, evidence that cognitive aspects of soccer performance can be enhanced through targeted training. Several studies investigated programs using video-based tactical scenarios, virtual reality simulations, and computer-based decision tasks, reporting improvements in players’ anticipatory skills, decision-making speed, and tactical awareness (Romeas et al., 2016).

Despite these encouraging findings, significant limitations exist. Most intervention studies are short-term, lack robust control groups, and involve small sample sizes. Moreover, very few studies track whether cognitive gains persist over time or translate into improved match performance. This gap is critical, as the goal of such interventions is not just improved test performance but tangible enhancements on the field.

Future research should focus on longitudinal intervention designs with larger participant groups and rigorous control conditions. Additionally, there is a need for more sophisticated outcome measures that combine cognitive testing with objective, in-game performance analytics, providing a fuller picture of how cognitive training impacts real-world soccer performance.

### 3.5 Visual search behavior and anticipation

Visual search behavior has emerged as a vital component of game intelligence, particularly in high-level soccer contexts where players must process complex visual environments rapidly. Studies consistently show that elite players use more efficient gaze patterns, focusing on relevant cues while filtering out extraneous information (Mann et al., 2007). This skill allows them to anticipate opponents’ movements, identify emerging tactical opportunities, and make proactive decisions under time pressure.

While visual search behavior is inherently linked to attentional control and working memory, its integration into broader models of executive functioning remains underdeveloped. Most studies treat visual search as a perceptual skill rather than exploring its interaction with higher-order cognitive processes. Future research could profitably investigate how visual search behaviors are supported by, and interact with, domain-general EFs, thereby contributing to a more unified model of cognitive-perceptual expertise in soccer.

### 3.6 Executive functions and injury prevention

An emerging but underexplored theme in the literature concerns the role of EFs in injury prevention. Some evidence suggests that stronger executive control may help players maintain attentional focus and avoid risky movements that increase injury likelihood (Scharfen and Memmert, 2021a,b). Cognitive overload, lapses in concentration, and diminished inhibitory control during fatigue or high-stress situations are hypothesized to contribute to injury risk, making EFs not only performance-enhancing but also health-preserving capacities.

Research in this area remains in its infancy. Few studies have directly measured EFs in relation to injury rates, and longitudinal research is especially lacking. Expanding this field could yield important insights into cognitive risk factors for injury and inform prevention strategies that integrate physical and cognitive training.

### 3.7 Neuropsychological assessment of executive functions

Among the included studies, a subset (e.g., Vestberg et al., 2012; Verburgh et al., 2014) relied on standardized neuropsychological tests to assess executive functions in controlled laboratory environments. These studies primarily measured working memory, cognitive flexibility, inhibitory control, and planning using established cognitive tasks such as the Stroop test, Trail Making Test, and digit span tasks. While these methods provide robust and replicable assessments of domain-general executive capacities, they may lack ecological validity, as they do not fully capture the dynamic and context-specific cognitive demands of real-game situations. However, such measures remain essential for isolating core cognitive functions and establishing baseline profiles of elite players’ cognitive abilities.

## 4 Discussion

The relationship between game intelligence and executive functions in soccer is an emerging field of inquiry, but findings from this review show that research on this topic remains surprisingly limited. One plausible explanation for this scarcity lies in the highly competitive and lucrative nature of the soccer industry. With top clubs operating in a billion-dollar ecosystem, proprietary knowledge on the assessment, interpretation, and training of game intelligence and executive functions is often viewed as a strategic advantage. As a result, clubs may refrain from sharing data, methods, or findings that could undermine their competitive edge.

The soccer industry operates in a highly commercialized environment, where winning matches and tournaments significantly impacts revenue streams from sponsorships, broadcasting rights, and merchandise sales. In this context, intellectual property related to player performance becomes a valuable asset. Top clubs often employ in-house research teams and cutting-edge technologies, such as wearable sensors and neurocognitive assessments, to analyze players’ cognitive and physical attributes. This creates a wealth of data that remains inaccessible to external researchers. Research collaborations between clubs and academic institutions are rare, as clubs are reluctant to disclose insights that could benefit their competitors. Clubs increasingly use private databases and machine learning algorithms to predict player performance, creating barriers for independent validation or replication of results.

Moreover, the recent findings by Bonetti et al. (2025) suggest that understanding game intelligence in elite soccer may require a broader perspective that includes personality profiling alongside cognitive assessment. By identifying consistent psychological traits among elite players, this research highlights the potential for integrated player development models that encompass both cognitive training and psychological skill development. Future studies should therefore consider multimodal approaches that evaluate how personality factors interact with executive functions to shape on-field behaviors and decision-making processes.

Additionally, planning, as a higher-order executive function, may serve as a bridge between cognitive abilities and strategic behavior on the field. The inclusion of planning in cognitive profiling could help explain how players not only adapt to immediate changes but also anticipate and structure play sequences over longer time frames, contributing to what is often described as tactical foresight in soccer. Given the strategic nature of high-level competition, future research should explicitly assess planning abilities in relation to game intelligence and explore how they interact with personality traits identified by Bonetti et al. (2025).

In this context, neuropsychological testing in controlled environments becomes an invaluable tool, as it allows researchers to bypass data restrictions while still capturing critical aspects of executive functioning. However, while neuropsychological measures contribute unique insights into the cognitive profiles of elite players, they must be understood as complementary to soccer-specific assessments, which better reflect the real-time, high-pressure decision-making that defines game intelligence. Future research should seek to integrate these approaches to overcome the limitations imposed by restricted access to in-game cognitive data.

Academic researchers often face challenges in accessing high-quality data and funding to conduct longitudinal studies on game intelligence and executive functions. This limitation is exacerbated by the lack of cooperation from professional clubs, which are key stakeholders in the field. Researchers have restricted access to elite players. Most studies rely on convenience samples, such as amateur or youth players, which may not accurately represent the cognitive demands of elite soccer. There may be a conflict of interest. Clubs that collaborate with researchers may impose restrictions on publishing findings, as highlighted by Vestberg et al. (2020), thereby limiting the broader applicability of results.

### 4.1 Limitations, further research and practical implications

Our methodological choices have led to limitations in the review process. Examples of choices that may have impacted the quality of the study is the selected databases and use of synonyms in the search string. These limitations may have been remedied by developing a transparent review protocol as used in traditional systematic reviews. Furthermore, a scoping review does not include a quality assessment of the reviewed studies, and it would have been interesting to view the included studies in light of quality assessment tools (Hong et al., 2018; Kmet et al., 2004).

A key challenge in this field is the lack of standardized tools for assessing both EFs and game intelligence. While some studies use neuropsychological tests, others rely on subjective evaluations, leading to inconsistent findings. Future research should focus on developing unified metrics to facilitate cross-study comparisons. In addition, the 12 included studies each provide unique perspectives on the interrelationship EFs and game intelligence in football. This complexity made it challenging to synthesize and compare the studies. Future studies should use more longitudinal designs to make it possible to examine relationships between the variables over time. Most studies to date are cross-sectional, limiting the ability to infer causal relationships between EFs and game intelligence. Longitudinal studies, such as those tracking players over multiple seasons, are needed to establish developmental trajectories. Randomized controlled trials (RCT) are indispensable for studying the relationship between EFs and game intelligence in football because they establish causal links, control bias, and ensure robust and valid findings. By leveraging RCT designs, researchers can develop evidence-based cognitive training interventions that enhance both EFs and game intelligence, ultimately improving performance on the field.

Research demonstrates that cognitive flexibility and working memory are strongly associated with tactical decision-making and adaptability in elite players (Vestberg et al., 2012; Radke et al., 2023). Training programs should integrate EFs exercises alongside technical and physical drills. This could be exercises like *dual task drills*: a combination of cognitive challenge with physical activities such as dribbling while solving pattern-recognition puzzles, *position-specific training*: tailor drills to match positional demands (e.g., defenders focusing on anticipatory skills, attackers on rapid decision-making), or *video-based decision training*: use match scenarios to enhance players’ ability to process dynamic game information.

Furthermore, studies like Krupitzer et al. (2022) highlight the efficacy of VR-based training in improving cognitive skills. Such tools simulate realistic, high-pressure scenarios, allowing players to practice decision-making without physical strain. VR platforms like CortexVR in training sessions could enhance game intelligence in a controlled environment. Also the use of eye-tracking systems to assess and train visual search behaviors seems to be beneficial for developing game intelligence, particularly for goalkeepers and defenders.

Team performance relies on the interplay of individual players’ EFs, as evidenced by Scharfen and Memmert (2021a,b). Better cognitive flexibility can enhance coordination during complex tactical movements, such as pressing or counter-attacking. Based on this, a practical implications may be to introduce team-based cognitive challenges to foster synchronization and train players to anticipate teammates’ decisions based on contextual cues. In addition, injury prevention strategies should incorporate EF training, as lapses in cognitive attention are linked to injury risks (Scharfen and Memmert, 2021a,b). Coaches should design drills that replicate high-stress match scenarios, emphasizing cognitive focus under fatigue and monitor EFs metrics as part of injury risk assessments in elite players.

The interplay between EFs and game intelligence is pivotal for success at the elite level of soccer. Practical applications, such as integrating EFs training into development programs and using advanced technologies like VR, offer promising ways to enhance performance. However, substantial gaps in knowledge remain, particularly regarding position-specific demands, the impact of cognitive load, and longitudinal EFs development. Future research must address these gaps through well-designed, longitudinal, and comparative studies. By deepening the understanding of how EFs shape game intelligence, researchers can help coaches, players, and practitioners optimize training and maximize elite performance.

## 5 Conclusion

This scoping review provides a systematic synthesis of empirical research on the relationship between executive functions (EFs) and game intelligence in adult elite soccer players. The review highlights that core EFs—working memory, cognitive flexibility, inhibitory control, perceptual anticipation and planning—are consistently linked to players’ capacity to process complex game situations, make rapid and adaptive decisions, and execute effective tactical behaviors under pressure. Furthermore, the evidence suggests that these cognitive processes may vary according to playing position, with offensive roles often demanding higher cognitive flexibility and attentional control, while defensive roles may prioritize anticipatory skills and pattern recognition.

The findings reveal that neuropsychological assessments remain essential tools in identifying individual cognitive profiles, despite challenges related to ecological validity and restricted access to in-game performance data due to commercial interests within elite football. Recent research, such as Bonetti et al. (2025), further expands the understanding of player performance by demonstrating how personality traits interact with cognitive abilities, suggesting that game intelligence emerges not solely from executive control but also from stable psychological characteristics that influence decision-making under stress.

However, the current literature is marked by methodological heterogeneity, with significant variations in how both EFs and game intelligence are conceptualized and measured. The dominance of cross-sectional designs and small sample sizes limits the ability to draw causal inferences or track the development of cognitive skills over time. Additionally, few studies have directly linked cognitive assessments to objective match performance metrics, leaving gaps in our understanding of how EFs translate into concrete actions on the field.

Future research should prioritize longitudinal designs, integrate neuropsychological and soccer-specific cognitive measures, and explore how cognitive training interventions might enhance both EFs and on-field performance. Moreover, combining cognitive profiling with personality assessments could provide a more holistic understanding of what distinguishes elite players, supporting more tailored approaches to player development and performance optimization.

By consolidating current knowledge and identifying critical gaps, this review contributes to the growing recognition of cognitive and psychological processes as fundamental components of expertise in elite soccer, while underscoring the need for more comprehensive, interdisciplinary research in this emerging field.

## Data Availability

The original contributions presented in the study are included in the article/supplementary material, further inquiries can be directed to the corresponding author.

## References

[ref1] Appelbaum L. G. Erickson G. (2018). Sports vision training: a review of the state-of-the-art in digital training techniques. Int. Rev. Sport Exerc. Psychol. 11, 160–189. doi: 10.1080/1750984X.2016.1266376

[ref2] Arksey H. O'Malley L. (2005). Scoping studies: towards a methodological framework. Int. J. Soc. Res. Methodol. 8, 19–32. doi: 10.1080/1364557032000119616

[ref3] Armendáriz M. L. P. Spyrou K. Alcaraz P. E. (2023). Match demands of female team sports: a scoping review. Biol. Sport 41, 175–199. doi: 10.5114/biolsport.2024.129476, PMID: 38188119 PMC10765441

[ref4] Beavan A. Spielmann J. Mayer J. Skorski S. Meyer T. Fransen J. (2020). The rise and fall of executive functions in high-level football players. Psychol. Sport Exerc. 49, 101677–101612. doi: 10.1016/j.psychsport.2020.101677, PMID: 32711397

[ref5] Bergkamp T. L. Frencken W. G. Niessen A. S. M. Meijer R. R. den Hartigh R. J. (2021). How soccer scouts identify talented players. Eur. J. Sport Sci. 22, 994–1004. doi: 10.1080/17461391.2021.1916081, PMID: 33858300

[ref6] Bonetti L. Vestberg T. Jafari R. Seghezzi D. Ingvar M. Kringelbach M. L. . (2025). Decoding the elite soccer player’s psychological profile. Psychol. Cogn. Sci. 122:e2415126122. doi: 10.1073/pnas.2415126122, PMID: 39808661 PMC11760505

[ref7] Braun V. Clarke V. Hayfield N. Davey L. Jenkinson E. (2023). “Doing reflexive thematic analysis” in Supporting research in counselling and psychotherapy: qualitative, quantitative, and mixed methods research (Cham: Springer International Publishing), 19–38.

[ref8] Brimmell J. Edwards E. J. Vaughan R. S. (2022). Executive function and visual attention in sport: a systematic review. Int. Rev. Sport Exerc. Psychol. 17, 1278–1311. doi: 10.1080/1750984X.2022.2145574, PMID: 40083416

[ref9] Clemente F. M. Ramirez-Campillo R. Nakamura F. Y. Sarmento H. (2021). Effects of high-intensity interval training in men soccer player’s physical fitness: a systematic review with meta-analysis of randomized-controlled and non-controlled trials. J. Sports Sci. 39, 1202–1222. doi: 10.1080/02640414.2020.1863644, PMID: 33423603

[ref10] Diamond A. (2013). Executive functions. Annu. Rev. Psychol. 64, 135–168. doi: 10.1146/annurev-psych-113011-143750, PMID: 23020641 PMC4084861

[ref11] Furley P. Memmert D. (2013). “Whom should I pass to?” the more options the more attentional guidance from working memory. PLoS One 8, 1–14. doi: 10.1371/journal.pone.0062278, PMID: 23658719 PMC3642109

[ref12] Furley P. Schütz L. M. Wood G. (2023). A critical review of research on executive functions in sport and exercise. Int. Rev. Sport Exerc. Psychol. 1-29, 1–29. doi: 10.1080/1750984X.2023.2217437, PMID: 40083416

[ref13] Habekost T. Ovesen J. Madsen J. B. (2024). Cognition in elite soccer players: a general model. Front. Psychol. 15, 1–15. doi: 10.3389/fpsyg.2024.1477262, PMID: 39723399 PMC11668572

[ref14] Heilmann F. Weinberg H. Wollny R. (2022). The impact of practicing open-vs. closed-skill sports on executive functions—a meta-analytic and systematic review with a focus on characteristics of sports. Brain Sci. 12:1071. doi: 10.3390/brainsci12081071, PMID: 36009134 PMC9406193

[ref15] Hong Q. N. Gonzalez-Reyes A. Pluye P. (2018). Improving the usefulness of a tool for appraising the quality of qualitative, quantitative and mixed methods studies, the mixed methods appraisal tool (MMAT). J. Eval. Clin. Pract. 24, 459–467. doi: 10.1111/jep.12884, PMID: 29464873

[ref16] Huijgen B. C. Elferink-Gemser M. T. Lemmink K. A. Visscher C. (2014). Multidimensional performance characteristics in selected and deselected talented soccer players. Eur. J. Sport Sci. 14, 2–10. doi: 10.1080/17461391.2012.725102 24533489

[ref17] Huijgen B. C. Leemhuis S. Kok N. M. Verburgh L. Oosterlaan J. Elferink-Gemser M. T. . (2015). Cognitive functions in elite and sub-elite youth soccer players aged 13 to 17 years. PLoS One 10:e0144580. doi: 10.1371/journal.pone.0144580, PMID: 26657073 PMC4691195

[ref18] Hyndman N. Lapsley I. Philippou C. (2024). Exploring a soccer society: dreams, themes and the beautiful game. Account. Audit. Account. J. 37, 433–453. doi: 10.1108/AAAJ-08-2023-6622

[ref19] Ilić I. (2016). “Structures and differences of the cognitive abilities of top handball, volleyball, basketball and soccer players” in Facta Universitatis. Series: Physical education and sport, 403–410.

[ref20] Jordet G. Aksum K. M. Pedersen D. N. Walvekar A. Trivedi A. McCall A. . (2020). Scanning, contextual factors, and association with performance in English premier league footballers: an investigation across a season. Front. Psychol. 11, 1–16. doi: 10.3389/fpsyg.2020.553813, PMID: 33123039 PMC7573254

[ref21] Julian R. Page R. M. Harper L. D. (2021). The effect of fixture congestion on performance during professional male soccer match-play: a systematic critical review with meta-analysis. Sports Med. 51, 255–273. doi: 10.1007/s40279-020-01359-9, PMID: 33068272 PMC7846542

[ref22] Kalén A. Bisagno E. Musculus L. Raab M. Pérez-Ferreirós A. Williams A. M. . (2021). The role of domain-specific and domain-general cognitive functions and skills in sports performance: a meta-analysis. Psychol. Bull. 147, 1290–1308. doi: 10.1037/bul0000355, PMID: 35404636

[ref23] Kermarrec G. Bossard C. (2020). Defensive soccer players` decision making: a naturalistic study. J. Cogn. Eng. Decision Mak. 8, 187–199. doi: 10.1177/1555343414527968

[ref24] Khaitovich K. F. (2023). The most popular sports in the world. Br. J. Glob. Ecol. Sustain. Dev. 17, 92–95.

[ref26] Kirkendall T. D. (2020). Evolution of soccer as a research topic. Prog. Cardiovasc. Dis. 63, 723–729. doi: 10.1016/j.pcad.2020.06.011, PMID: 32599029

[ref27] Klatt S. Nerb J. (2021). Position-specific attentional skills in team sports: a comparison between defensive and offensive football players. Appl. Sci. 11, 1–13. doi: 10.3390/app11135896, PMID: 40053772

[ref28] Kmet L. Lee R. Cook L. (2004). Standard quality assessment criteria for evaluating primary research papers from a variety of fields. Edmonton, Alberta, Canada: Alberta Heritage Foundation for Medical Research.

[ref29] Krupitzer C. Naber J. Stauffert J. P. Mayer J. Spielmann J. Ehmann P. . (2022). CortexVR: immersive analysis and training of cognitive executive functions of soccer players using virtual reality and machine learning. Front. Psychol. 13:754732. doi: 10.3389/fpsyg.2022.754732, PMID: 36081714 PMC9446155

[ref30] Krustrup P. Parnell D. (2019). Football as medicine: prescribing football for global health promotion. Abingdon: Routledge.

[ref31] Larkin P. O’Connor D. (2017). Talent identification and recruitment in youth soccer: recruiter’s perceptions of the key attributes for player recruitment. PLoS One 12:e0175716-15. doi: 10.1371/journal.pone.0175716, PMID: 28419175 PMC5395184

[ref33] Mangine G. T. Hoffman J. R. Wells A. J. Gonzalez A. M. Rogowski J. P. Townsend J. R. . (2014). Visual tracking speed is related to basketball-specific measures of performance in NBA players. J. Strength Cond. Res. 28, 2406–2414. doi: 10.1519/JSC.0000000000000550, PMID: 24875429

[ref34] Mann D. L. Causer J. Nakamoto H. Runswick O. R. (2019). “Visual search behaviours in expert perceptual judgements” in Anticipation and decision making in sport. eds. Williams A. M. Jackson R. (London: Routledge), 59–78.

[ref35] Mann D. T. Williams A. M. Ward P. Janelle C. M. (2007). Perceptual-cognitive expertise in sport: a meta-analysis. J. Sport Exerc. Psychol. 29, 457–478. doi: 10.1123/jsep.29.4.457, PMID: 17968048

[ref37] Miyake A. Friedman N. P. (2012). The nature and organization of individual differences in executive functions: four general conclusions. Curr. Dir. Psychol. Sci. 21, 8–14. doi: 10.1177/0963721411429458, PMID: 22773897 PMC3388901

[ref38] Moen F. Hrozanova M. Stiles T. (2018). The effects of perceptual-cognitive training with neurotracker on executive brain functions among elite athletes. Cogent Psychol. 5, 1–13. doi: 10.1080/23311908.2018.1544105, PMID: 40083416

[ref9001] Moher D. Liberati A. Tetzlaff J. Altman D. G., and Prisma Group. (2010). Preferred reporting items for systematic reviews and meta-analyses: the PRISMA statement. International journal of surgery, 8, 336–341. doi: 10.1016/j.ijsu.2010.02.007 20171303

[ref39] Murr D. Feichtinger P. Larkin P. O’Connor D. Höner O. (2018). Psychological talent predictors in youth soccer: a systematic review of the prognostic relevance of psychomotor, perceptual-cognitive and personality-related factors. PLoS One 13:e0205337. doi: 10.1371/journal.pone.0205337, PMID: 30321221 PMC6188900

[ref41] Nicholson B. Dinsdale A. Jones B. Till K. (2022). The training of medium-to long-distance sprint performance in football code athletes: a systematic review and meta-analysis. Sports Med. 52, 257–286. doi: 10.1007/s40279-021-01552-4, PMID: 34499339 PMC8803780

[ref42] Nunez F. J. Ona A. Raya A. Bilbao A. (2009). Differences between expert and novice soccer players when using movement precues to shoot a penalty kick. Perceptual Motor Skills 108, 139–148. doi: 10.2466/pms.108.1.139-148, PMID: 19425456

[ref43] Nunez F. J. Ona A. Raya A. Bilbao A. (2010). Effects of providing advance cues during a soccer penalty kick on the kicker’s rate of success. Perceptual Motor Skills 111, 749–760. doi: 10.2466/05.24.25.Pms.111.6.749-760 21319614

[ref44] Owoeye O. B. VanderWey M. J. Pike I. (2020). Reducing injuries in soccer (football): an umbrella review of best evidence across the epidemiological framework for prevention. Sports Med. 6:46. doi: 10.1186/s40798-020-00274-7, PMID: 32955626 PMC7505904

[ref45] Radke L. Mertens A. Spielmann J. Mayer J. (2023). Being ahead of the game—the association between executive functions and football performance in high-level football players. Ger. J. Exerc. Sport Res. 53, 288–300. doi: 10.1007/s12662-023-00885-8

[ref46] Roberts S. J. McRobert A. P. Lewis C. J. Reeves M. J. (2019). Establishing consensus of position-specific predictors for elite youth soccer in England. Sci. Med. Footb. 3, 205–213. doi: 10.1080/24733938.2019.1581369

[ref9002] Roca A. Ford P. R. Williams A. M. (2013). The processes underlying ‘game intelligence’ skills in soccer players. In Nunome H. Drust B. Dawson B. (Eds.), Science and football VII (pp. 255–260). Abingdon, Oxon: Routledge.

[ref47] Roca A. Ford P. R. Memmert D. (2018, 2018). Creative decision making and visual search behavior in skilled soccer players. PLoS One 13, 1–11. doi: 10.1371/journal.pone.0199381, PMID: 29990320 PMC6039007

[ref48] Romeas T. Guldner A. Faubert J. (2016). 3D-multiple object tracking training task improves passing decision-making accuracy in soccer players. Psychol. Sport Exerc. 22, 1–9. doi: 10.1016/j.psychsport.2015.06.002

[ref9003] Sakamoto S. Takeuchi H. Ihara N. Ligao B. Suzukawa K. (2018). Possible requirement of executive functions for high performance in soccer. PloS one, 13:e0201871. doi: 10.1371/journal.pone.0201871 30133483 PMC6104941

[ref49] Sanchez-Sanchez J. Rodriguez-Fernandez A. Granacher U. Afonso J. Ramirez-Campillo R. (2024). Plyometric jump training effects on maximal strength in soccer players: a systematic review with Meta-analysis of randomized-controlled studies. Sports Med. 10:52. doi: 10.1186/s40798-024-00720-w, PMID: 38727944 PMC11087442

[ref50] Sarmento H. Clemente F. M. Araújo D. Davids K. McRobert A. Figueiredo A. (2018). What performance analysts need to know about research trends in association football (2012–2016): a systematic review. Sports Med. 48, 799–836. doi: 10.1007/s40279-017-0836-6, PMID: 29243038

[ref51] Savelsbergh G. J. P. Van der Kamp J. Williams A. M. Ward P. (2005). Anticipation and visual search behaviour in expert soccer goalkeepers. Egnonomics 48, 1686–1697. doi: 10.1080/00140130500101346, PMID: 16338733

[ref52] Savelsbergh G. J. P. Williams A. M. Van Der Kamp J. Warrd P. (2002). Visual search, anticipation and expertise in soccer goalkeepers. J. Sports Sci. 20, 279–287. doi: 10.1080/026404102317284826, PMID: 11999482

[ref53] Scharfen H. E. Memmert D. (2019). Measurement of cognitive functions in experts and elite athletes: a meta-analytic review. Appl. Cogn. Psychol. 33, 843–860. doi: 10.1002/acp.3526

[ref54] Scharfen H. E. Memmert D. (2021a). Cognitive training in elite soccer players: evidence of narrow, but not broad transfer to visual and executive function. Ger. J. Exerc. Sport Res. 51, 135–145. doi: 10.1007/s12662-020-00699-y

[ref55] Scharfen H. E. Memmert D. (2021b). Fundamental relationships of executive functions and physiological abilities with game intelligence, game time and injuries in elite soccer players. Appl. Cogn. Psychol. 35, 1535–1546. doi: 10.1002/acp.3886

[ref56] Šetić R. Kolenović-Dapo J. Talović M. (2017). Validation study of the tactical-technical and social competencies of football players scale. Sportski Logos 15, 11–18.

[ref57] Šetić R. Kolenović-Dapo J. Talović M. (2019). Validation scale study for assessment of tactical and technical competencies among footballers – assessment by trainers. Homo Sporticus 2, 29–35.

[ref58] Strauss E. Sherman E. M. S. Spreen O. (2006). A compendium of neurpsychological tests Admnistration, norms and commentary. Oxford: Oxford University Press.

[ref59] Temple V. A. Crane J. R. (2018). A systematic review of drop-out from organized soccer among children and adolescents. Junior Youth Grassroots Football Culture 17, 856–881. doi: 10.1080/14660970.2015.1100901

[ref60] Teoldo I. Guilherme J. Garganta J. (2021). Football intelligence: training and tactics for soccer success. Abingdon, Oxon and New York, NY: Routledge: Routledge.

[ref61] Thapa R. K. Narvariya P. Weldon A. Talukdar K. Ramirez-Campillo R. (2022). Can complex contrast training interventions improve aerobic endurance, maximal strength, and repeated sprint ability in soccer players? A systematic review and meta-analysis. Montenegrin J. Sports Sci. Med. 11, 45–55. doi: 10.26773/mjssm.220906

[ref62] Tovar J. (2021). Soccer, world war II and coronavirus: a comparative analysis of how the sport shut down. Soccer Soc. 22, 66–74. doi: 10.1080/14660970.2020.1755270

[ref63] van de Hoef P. A. Brauers J. J. van Smeden M. Backx F. J. Brink M. S. (2020). The effects of lower-extremity plyometric training on soccer-specific outcomes in adult male soccer players: a systematic review and meta-analysis. Int. J. Sports Physiol. Perform. 15, 3–17. doi: 10.1123/ijspp.2019-0565, PMID: 31810063

[ref64] Verburgh L. Scherder E. J. van Lange P. A. Oosterlaan J. (2014). Executive functioning in highly talented soccer players. PLoS One 9:e91254. doi: 10.1371/journal.pone.0091254, PMID: 24632735 PMC3954684

[ref65] Vestberg T. Gustafson R. Maurex L. Ingvar M. Petrovic P. (2012). Executive functions predict the success of top-soccer players. PLoS One 7, e34731–e34735. doi: 10.1371/journal.pone.0034731, PMID: 22496850 PMC3319604

[ref66] Vestberg T. Jafari R. Almeida R. Maurex L. Ingvar M. Petrovic P. (2020). Level of play and coach-rated game intelligence are related to performance on design fluency in elite soccer players. Sci. Rep. 10, 9852–9810. doi: 10.1038/s41598-020-66180-w, PMID: 32587269 PMC7316809

[ref67] Ward P. Williams A. M. (2003). Perceptual and cognitive skill development in soccer: the multidimensional nature of expert performance. J. Sport Exerc. Psychol. 25, 93–111. doi: 10.1123/jsep.25.1.93

[ref69] Williams A. M. Hodges N. J. (2005). Practice, instruction and skill acquisition in soccer: challenging tradition. J. Sports Sci. 23, 637–650. doi: 10.1080/02640410400021328, PMID: 16195012

[ref70] Williams A. M. Jackson R. C. (2019). Anticipation in sport: fifty years on, what have we learned and what research still needs to be undertaken? Psychol. Sport Exerc. 42, 16–24. doi: 10.1016/j.psychsport.2018.11.014

[ref71] Zhang J. J. Pitts B. G. (2018). The global football industry: marketing perspectives. London: Routledge.

[ref72] Zhao J. Gu Q. Zhao S. Mao J. (2022). Effects of video-based training on anticipation and decision-making in football players: a systematic review. Front. Hum. Neurosci. 16:945067. doi: 10.3389/fnhum.2022.945067, PMID: 36438631 PMC9686440

